# *cyclo*-Tetra-μ-malato-κ^16^ *O*,*O*′,*O*′′:*O*′′′-tetra­kis[bis­(1*H*-benzimidazole-κ*N* ^3^)cobalt(II)] eicosa­hydrate

**DOI:** 10.1107/S1600536808006715

**Published:** 2008-03-14

**Authors:** Jun-Hua Li, Jing-Jing Nie, Jian-Rong Su, Duan-Jun Xu

**Affiliations:** a Department of Chemistry, Zhejiang University, People’s Republic of China

## Abstract

The title compound, [Co_4_(C_4_H_4_O_5_)_4_(C_7_H_6_N_2_)_8_]·20H_2_O, consists of tetra­nuclear Co^II^ complexes and disordered uncoordinated water mol­ecules. The tetra­meric complex mol­ecule has 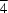 symmetry. While two benzimidazole mol­ecules and a tridentate malate dianion coordinate a Co^II^ ion, the carboxylate O atom from an adjacent malate dianion bridges the Co^II^ ions to complete a distorted octa­hedral coordination geometry. The tridentate malate dianion chelates the Co^II^ ion, and the chelate six- and five-membered rings show half-chair and envelope configurations, respectively. A face-to-face separation of 3.494 (9) Å between parallel benzimidazole ligands indicates the existence of π–π stacking between adjacent complexes. The crystal structure also involves N—H⋯O and O—H⋯O hydrogen bonds.

## Related literature

For general background, see: Deisenhofer & Michel (1989 ▶); Su & Xu (2004 ▶); Liu *et al.* (2004 ▶); Li *et al.* (2005 ▶). For related structures, see: Nie *et al.* (2002 ▶);
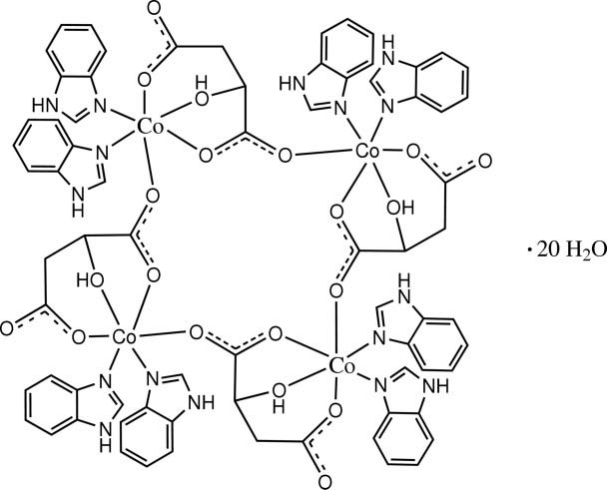


## Experimental

### 

#### Crystal data

[Co_4_(C_4_H_4_O_5_)_4_(C_7_H_6_N_2_)_8_]·20H_2_O
*M* *_r_* = 2069.43
Tetragonal, 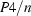

*a* = 20.230 (2) Å
*c* = 11.6203 (12) Å
*V* = 4755.6 (8) Å^3^
*Z* = 2
Mo *K*α radiation
μ = 0.78 mm^−1^
*T* = 295 (2) K
0.35 × 0.30 × 0.22 mm

#### Data collection

Rigaku R-AXIS RAPID IP diffractometer
Absorption correction: multi-scan (*ABSCOR*; Higashi, 1995 ▶) *T*_min_ = 0.748, *T*_max_ = 0.840
29983 measured reflections
4053 independent reflections
3191 reflections with *I* > 2σ(*I*)
*R*_int_ = 0.075

#### Refinement

*R*[*F* ^2^ > 2σ(*F* ^2^)] = 0.076
*wR*(*F* ^2^) = 0.190
*S* = 1.15
4053 reflections
294 parameters
H-atom parameters constrained
Δρ_max_ = 0.43 e Å^−3^
Δρ_min_ = −0.31 e Å^−3^

### 

Data collection: *PROCESS-AUTO* (Rigaku, 1998 ▶); cell refinement: *PROCESS-AUTO*; data reduction: *CrystalStructure* (Rigaku/MSC, 2002 ▶); program(s) used to solve structure: *SIR92* (Altomare *et al.*, 1993 ▶); program(s) used to refine structure: *SHELXL97* (Sheldrick, 2008 ▶); molecular graphics: *ORTEP-3 for Windows* (Farrugia, 1997 ▶); software used to prepare material for publication: *WinGX* (Farrugia, 1999 ▶).

## Supplementary Material

Crystal structure: contains datablocks I, global. DOI: 10.1107/S1600536808006715/ng2434sup1.cif

Structure factors: contains datablocks I. DOI: 10.1107/S1600536808006715/ng2434Isup2.hkl

Additional supplementary materials:  crystallographic information; 3D view; checkCIF report

## Figures and Tables

**Table 1:** Selected bond lengths (Å)

Co—N13	2.078 (4)
Co—N23	2.075 (4)
Co—O1	2.112 (4)
Co—O3	2.150 (4)
Co—O4	2.169 (3)
Co—O5^i^	2.101 (4)

Symmetry code: (i) 
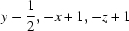
.

**Table 2:** Hydrogen-bond geometry (Å, °)

*D*—H⋯*A*	*D*—H	H⋯*A*	*D*⋯*A*	*D*—H⋯*A*
N11—H11⋯O3*WA*	0.86	2.18	3.00 (4)	159
N11—H11⋯O5*WA*^ii^	0.86	2.10	2.93 (4)	163
N21—H21⋯O1^iii^	0.86	2.57	3.258 (7)	138
N21—H21⋯O2^iii^	0.86	2.07	2.901 (8)	163
O3—H3*A*⋯O4^i^	0.92	1.78	2.645 (5)	155

Symmetry codes: (i) 
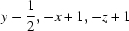
; (ii) 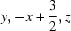; (iii) 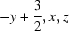.

**Fig. 1.**
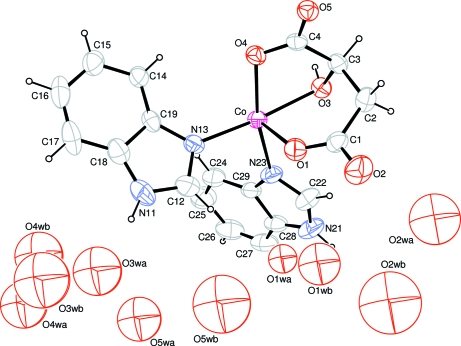
The structure of the title compound in an asymmetric unit with 30% probability displacement (arbitrary spheres for H atoms).

**Fig. 2.**
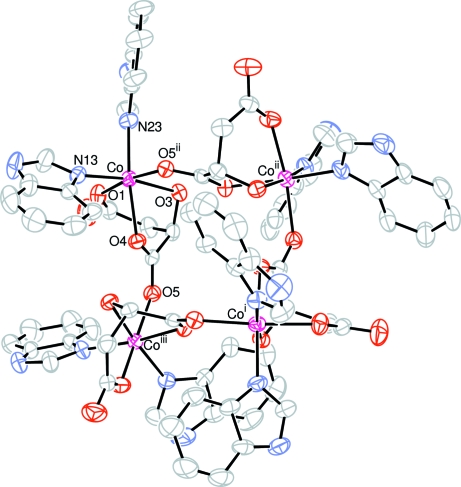
The tetranuclear molecular structure of the title complex [symmetry codes: (i) 1/2 - x,3/2 - y,z; (ii) -1/2 + y,1 - x,1 - z; (iii) 1 - y,1/2 + x,1 - z].

**Fig. 3.**
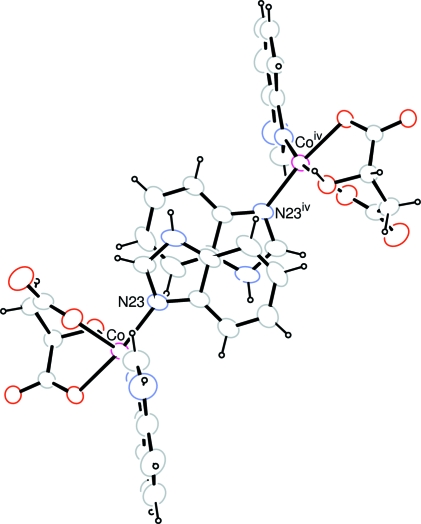
A diagram showing π-π stacking [symmetry code: (iv) 1 - x,1 - y,1 - z].

## supplementary crystallographic information

### Comment

As π-π stacking between aromatic rings plays an important role in electron transfer process in some biological system (Deisenhofer & Michel, 1989), π-π stacking has attracted our much attention in past years. A series of metal complexes with benzimidazole (bzim) ligand has been prepared and their crystal structures have been determined in our laboratory (Su & Xu, 2004; Liu *et al.*, 2004; Li *et al.*, 2005). As part of our ongoing investigation on the nature of π-π stacking, the title Co^II^ complex incorporating bzim ligand has been prepared and its crystal structure is reported here.

The structure in an asymmetric unit is shown in Fig. 1, and the molecular structure of the tetrameric complex is shown in Fig. 2. The tetranuclear complex has a -4 symmetry. In the asymmetric unit the Co^II^ ion is coordinated by two bzim ligands and a tridentate malate dianion, and the carboxyl O5 atom of malate dianion from the adjacent asymmetric unit bridges to the Co^II^ ion to complete the distorted octahedral coordination (Table 1). The uncoordinated carboxyl O2 atom links with the bzim ligand of the adjacent complex *via* N—H···O hydrogen bonding (Table 2). The tridentate malate dianion chelates the Co^II^ ion, the chelating six-membered ring and five-membered ring show half-chair and envelope configuration, respectively. Within the malate dianion the C1—C2—C3—C4 torsion angle of 58.9 (6)° is close to 53.0 (3)° found in the crystal structure of a free malic acid (Nie *et al.*, 2002).

Partially overlapped arrangement is observed between parallel bzim ligands of adjacent complexes (Fig. 3), the face-to-face separation of 3.494 (9) Å between N23-phen and N23^iv^-phen ligands indicates the existence of π-π stacking [symmetry code: (iv) 1 - *x*,1 - *y*,1 - *z*]. Lattice water molecules are disorderly distributed in the roomy space between complexes.

### Experimental

An ethanol solution (5 ml) of bzim (0.24 g, 2 mmol) was mixed with an aqueous solution (10 ml) containing cobalt(II) acetate tetrahydrate (0.24 g, 1 mmol), D,*L*-malic acid (0.13 g, 1 mmol) and sodium carbonate (0.10 g, 1 mmol). The mixture was refluxed for 4 h and filtered after cooling to room temperature. Single crystals of the title compound were obtained after one week from the filtrate.

### Refinement

Lattice water molecules are disordered in the crystal structure. Disordered lattice water O atoms were refined isotropically with the fixed occupancies of 1/2, and H atoms of disordered water molecules were not included in the refinement. Hydroxyl H atom was located in a difference Fourier map and refined as riding in its as-found relative position with *U*_iso_(H) = 1.2*U*_eq_(O). Other H atoms were placed in calculated positions with C—H = 0.93 (aromatic), 0.97 Å (methylene) and N—H = 0.86 Å, and refined in riding mode with *U*_iso_(H) = 1.2*U*_eq_(C,*N*).

### Crystal data

**Table:**

[Co_4_(C_4_H_4_O_5_)_4_(C_7_H_6_N_2_)_8_]·20H_2_O	*Z* = 2
*M**_r_* = 2069.43	*F*_000_ = 2152
Tetragonal, *P*4/*n*	*D*_x_ = 1.445 Mg m^−^^3^
Hall symbol: -P 4a	Mo *K*α radiation λ = 0.71069 Å
*a* = 20.230 (2) Å	Cell parameters from 8672 reflections
*b* = 20.230 Å	θ = 2.0–24.0º
*c* = 11.6203 (12) Å	µ = 0.78 mm^−^^1^
α = 90º	*T* = 295 (2) K
β = 90º	Prism, pink
γ = 90º	0.35 × 0.30 × 0.22 mm
*V* = 4755.6 (8) Å^3^	

### Data collection

**Table:**

Rigaku R-AXIS RAPID IP diffractometer	4053 independent reflections
Radiation source: fine-focus sealed tube	3191 reflections with *I* > 2σ(*I*)
Monochromator: graphite	*R*_int_ = 0.075
Detector resolution: 10.00 pixels mm^-1^	θ_max_ = 25.0º
*T* = 295(2) K	θ_min_ = 1.4º
ω scans	*h* = −22→24
Absorption correction: multi-scan(ABSCOR; Higashi, 1995)	*k* = −23→21
*T*_min_ = 0.748, *T*_max_ = 0.840	*l* = −12→12
29983 measured reflections	

### Refinement

**Table:**

Refinement on *F*^2^	Hydrogen site location: inferred from neighbouring sites
Least-squares matrix: full	H-atom parameters constrained
*R*[*F*^2^ > 2σ(*F*^2^)] = 0.076	*w* = 1/[σ^2^(*F*_o_^2^) + (0.0761*P*)^2^ + 7.4753*P*] where *P* = (*F*_o_^2^ + 2*F*_c_^2^)/3
*wR*(*F*^2^) = 0.190	(Δ/σ)_max_ = 0.001
*S* = 1.15	Δρ_max_ = 0.43 e Å^−^^3^
4053 reflections	Δρ_min_ = −0.31 e Å^−^^3^
294 parameters	Extinction correction: SHELXL97 (Sheldrick, 2008), Fc^*^=kFc[1+0.001xFc^2^λ^3^/sin(2θ)]^-1/4^
Primary atom site location: structure-invariant direct methods	Extinction coefficient: 0.0022 (4)
Secondary atom site location: difference Fourier map	

### Special details

**Table:**

Geometry. All e.s.d.'s (except the e.s.d. in the dihedral angle between two l.s. planes) are estimated using the full covariance matrix. The cell e.s.d.'s are taken into account individually in the estimation of e.s.d.'s in distances, angles and torsion angles; correlations between e.s.d.'s in cell parameters are only used when they are defined by crystal symmetry. An approximate (isotropic) treatment of cell e.s.d.'s is used for estimating e.s.d.'s involving l.s. planes.
Refinement. Refinement of *F*^2^ against ALL reflections. The weighted *R*-factor *wR* and goodness of fit *S* are based on *F*^2^, conventional *R*-factors *R* are based on *F*, with *F* set to zero for negative *F*^2^. The threshold expression of *F*^2^ > σ(*F*^2^) is used only for calculating *R*-factors(gt) *etc*. and is not relevant to the choice of reflections for refinement. *R*-factors based on *F*^2^ are statistically about twice as large as those based on *F*, and *R*- factors based on ALL data will be even larger.

### Fractional atomic coordinates and isotropic or equivalent isotropic displacement parameters (Å^2^)

**Table:**

	*x*	*y*	*z*	*U*_iso_*/*U*_eq_	Occ. (<1)
Co	0.43554 (3)	0.71743 (3)	0.55558 (6)	0.0519 (3)	
N11	0.5316 (3)	0.8011 (3)	0.8403 (6)	0.106 (2)	
H11	0.5680	0.8134	0.8721	0.128*	
N13	0.4649 (2)	0.7567 (2)	0.7126 (4)	0.0602 (11)	
N21	0.5804 (3)	0.5747 (3)	0.4877 (6)	0.0909 (18)	
H21	0.6114	0.5610	0.4429	0.109*	
N23	0.5019 (2)	0.6392 (2)	0.5566 (4)	0.0667 (12)	
O1	0.50329 (18)	0.7760 (2)	0.4621 (4)	0.0691 (11)	
O2	0.5563 (2)	0.8172 (3)	0.3159 (4)	0.1038 (16)	
O3	0.39943 (17)	0.69288 (15)	0.3869 (3)	0.0571 (9)	
H3A	0.3602	0.6703	0.3976	0.068*	
O4	0.36459 (17)	0.79509 (16)	0.5193 (3)	0.0556 (9)	
O5	0.33611 (16)	0.85780 (16)	0.3690 (3)	0.0562 (9)	
C1	0.5066 (3)	0.7899 (3)	0.3585 (6)	0.0628 (14)	
C2	0.4512 (3)	0.7765 (3)	0.2754 (5)	0.0712 (16)	
H2A	0.4652	0.7422	0.2225	0.085*	
H2B	0.4434	0.8162	0.2306	0.085*	
C3	0.3868 (2)	0.7556 (2)	0.3297 (5)	0.0570 (13)	
H3	0.3541	0.7486	0.2688	0.068*	
C4	0.3602 (2)	0.8070 (2)	0.4135 (5)	0.0506 (12)	
C12	0.5260 (3)	0.7690 (4)	0.7395 (7)	0.093 (2)	
H12	0.5619	0.7569	0.6941	0.111*	
C14	0.3604 (3)	0.7854 (3)	0.8185 (5)	0.0652 (15)	
H14	0.3313	0.7661	0.7663	0.078*	
C15	0.3380 (4)	0.8176 (4)	0.9149 (6)	0.088 (2)	
H15	0.2927	0.8205	0.9272	0.106*	
C16	0.3800 (6)	0.8455 (4)	0.9937 (7)	0.104 (2)	
H16	0.3624	0.8663	1.0581	0.125*	
C17	0.4469 (6)	0.8436 (4)	0.9804 (6)	0.110 (3)	
H17	0.4756	0.8629	1.0331	0.132*	
C18	0.4696 (4)	0.8110 (3)	0.8834 (6)	0.0838 (18)	
C19	0.4273 (3)	0.7829 (2)	0.8026 (5)	0.0584 (13)	
C22	0.5461 (3)	0.6295 (3)	0.4751 (7)	0.086 (2)	
H22	0.5526	0.6587	0.4144	0.103*	
C24	0.4745 (3)	0.5679 (3)	0.7273 (6)	0.086 (2)	
H24	0.4417	0.5951	0.7572	0.104*	
C25	0.4908 (4)	0.5093 (4)	0.7806 (7)	0.109 (3)	
H25	0.4695	0.4967	0.8480	0.131*	
C26	0.5408 (4)	0.4680 (4)	0.7312 (9)	0.108 (3)	
H26	0.5506	0.4280	0.7669	0.130*	
C27	0.5738 (3)	0.4843 (4)	0.6366 (8)	0.097 (2)	
H27	0.6064	0.4568	0.6065	0.116*	
C28	0.5580 (3)	0.5432 (3)	0.5846 (6)	0.0707 (17)	
C29	0.5082 (2)	0.5850 (3)	0.6283 (6)	0.0637 (15)	
O1WA	0.6498 (6)	0.7660 (6)	0.5347 (11)	0.111 (4)*	0.50
O1WB	0.6657 (13)	0.7678 (12)	0.450 (2)	0.232 (10)*	0.50
O2WA	0.612 (2)	0.656 (2)	0.164 (4)	0.382 (18)*	0.50
O2WB	0.724 (5)	0.683 (3)	0.306 (5)	0.55 (4)*	0.50
O3WA	0.6433 (16)	0.8792 (19)	0.941 (3)	0.294 (13)*	0.50
O3WB	0.656 (2)	0.954 (2)	1.036 (3)	0.358 (17)*	0.50
O4WA	0.6559 (16)	0.7823 (16)	1.177 (3)	0.306 (14)*	0.50
O4WB	0.6030 (17)	0.8444 (17)	1.094 (3)	0.305 (13)*	0.50
O5WA	0.6861 (19)	0.6634 (15)	0.943 (2)	0.294 (13)*	0.50
O5WB	0.688 (3)	0.692 (3)	0.719 (4)	0.49 (3)*	0.50

### Atomic displacement parameters (Å^2^)

**Table:**

	*U*^11^	*U*^22^	*U*^33^	*U*^12^	*U*^13^	*U*^23^
Co	0.0465 (4)	0.0514 (4)	0.0579 (5)	0.0028 (3)	−0.0040 (3)	−0.0070 (3)
N11	0.092 (5)	0.139 (6)	0.088 (5)	−0.022 (4)	−0.033 (4)	−0.028 (4)
N13	0.053 (3)	0.066 (3)	0.061 (3)	0.003 (2)	−0.011 (2)	−0.006 (2)
N21	0.065 (3)	0.085 (4)	0.122 (5)	0.016 (3)	0.020 (3)	−0.020 (4)
N23	0.050 (3)	0.068 (3)	0.082 (3)	0.013 (2)	0.002 (2)	−0.009 (3)
O1	0.059 (2)	0.080 (3)	0.068 (3)	−0.0198 (18)	−0.0013 (19)	−0.005 (2)
O2	0.085 (3)	0.119 (4)	0.108 (4)	−0.038 (3)	0.019 (3)	−0.004 (3)
O3	0.061 (2)	0.0489 (19)	0.061 (2)	−0.0063 (15)	−0.0033 (17)	−0.0063 (16)
O4	0.060 (2)	0.0530 (19)	0.054 (2)	0.0098 (15)	−0.0076 (17)	−0.0001 (16)
O5	0.055 (2)	0.057 (2)	0.056 (2)	−0.0009 (15)	−0.0015 (17)	0.0061 (17)
C1	0.053 (3)	0.063 (3)	0.072 (4)	−0.003 (2)	0.006 (3)	−0.017 (3)
C2	0.077 (4)	0.074 (4)	0.062 (4)	−0.008 (3)	0.010 (3)	−0.010 (3)
C3	0.056 (3)	0.060 (3)	0.055 (3)	−0.006 (2)	−0.009 (2)	−0.009 (3)
C4	0.037 (2)	0.052 (3)	0.063 (4)	−0.005 (2)	−0.007 (2)	−0.003 (2)
C12	0.064 (4)	0.122 (6)	0.093 (6)	0.000 (4)	−0.014 (4)	−0.022 (5)
C14	0.073 (4)	0.071 (4)	0.052 (4)	0.014 (3)	−0.004 (3)	0.003 (3)
C15	0.104 (5)	0.099 (5)	0.062 (5)	0.024 (4)	0.007 (4)	0.011 (4)
C16	0.154 (8)	0.108 (6)	0.050 (4)	0.008 (5)	0.006 (5)	−0.008 (4)
C17	0.158 (8)	0.116 (6)	0.056 (5)	−0.033 (6)	−0.008 (5)	−0.023 (4)
C18	0.091 (5)	0.090 (5)	0.071 (5)	−0.014 (4)	−0.011 (4)	−0.006 (4)
C19	0.076 (4)	0.052 (3)	0.047 (3)	0.002 (2)	−0.012 (3)	0.004 (2)
C22	0.073 (4)	0.079 (4)	0.105 (6)	0.011 (3)	0.022 (4)	−0.011 (4)
C24	0.075 (4)	0.095 (5)	0.089 (5)	0.030 (3)	−0.007 (4)	0.008 (4)
C25	0.102 (6)	0.114 (6)	0.111 (6)	0.034 (5)	−0.001 (5)	0.030 (5)
C26	0.093 (6)	0.092 (5)	0.140 (8)	0.028 (4)	−0.024 (5)	0.027 (5)
C27	0.064 (4)	0.081 (5)	0.144 (8)	0.020 (3)	−0.020 (5)	−0.019 (5)
C28	0.056 (3)	0.059 (3)	0.098 (5)	0.016 (3)	−0.016 (3)	−0.017 (3)
C29	0.046 (3)	0.073 (4)	0.072 (4)	0.011 (2)	−0.012 (3)	−0.013 (3)

### Geometric parameters (Å, °)

**Table:**

Co—N13	2.078 (4)	C2—H2B	0.9700
Co—N23	2.075 (4)	C3—C4	1.523 (7)
Co—O1	2.112 (4)	C3—H3	0.9800
Co—O3	2.150 (4)	C12—H12	0.9300
Co—O4	2.169 (3)	C14—C19	1.367 (8)
Co—O5^i^	2.101 (4)	C14—C15	1.373 (8)
N11—C12	1.344 (9)	C14—H14	0.9300
N11—C18	1.366 (9)	C15—C16	1.371 (11)
N11—H11	0.8600	C15—H15	0.9300
N13—C12	1.298 (7)	C16—C17	1.363 (12)
N13—C19	1.396 (7)	C16—H16	0.9300
N21—C22	1.316 (8)	C17—C18	1.382 (11)
N21—C28	1.370 (9)	C17—H17	0.9300
N21—H21	0.8600	C18—C19	1.392 (8)
N23—C22	1.318 (8)	C22—H22	0.9300
N23—C29	1.382 (7)	C24—C25	1.377 (9)
O1—C1	1.239 (7)	C24—C29	1.382 (9)
O2—C1	1.250 (7)	C24—H24	0.9300
O3—C3	1.454 (6)	C25—C26	1.432 (11)
O3—H3A	0.9247	C25—H25	0.9300
O4—C4	1.256 (6)	C26—C27	1.328 (11)
O5—C4	1.250 (6)	C26—H26	0.9300
C1—C2	1.504 (8)	C27—C28	1.375 (10)
C2—C3	1.507 (7)	C27—H27	0.9300
C2—H2A	0.9700	C28—C29	1.409 (7)
			
N23—Co—N13	95.85 (18)	C4—C3—H3	108.8
N23—Co—O5^i^	95.05 (16)	O5—C4—O4	126.1 (5)
N13—Co—O5^i^	92.60 (16)	O5—C4—C3	115.8 (5)
N23—Co—O1	90.65 (18)	O4—C4—C3	118.1 (4)
N13—Co—O1	92.93 (17)	N13—C12—N11	112.5 (6)
O5^i^—Co—O1	171.61 (15)	N13—C12—H12	123.8
N23—Co—O3	92.79 (16)	N11—C12—H12	123.8
N13—Co—O3	170.57 (15)	C19—C14—C15	117.0 (6)
O5^i^—Co—O3	90.39 (13)	C19—C14—H14	121.5
O1—Co—O3	83.18 (14)	C15—C14—H14	121.5
N23—Co—O4	168.94 (18)	C16—C15—C14	122.4 (7)
N13—Co—O4	94.74 (15)	C16—C15—H15	118.8
O5^i^—Co—O4	87.65 (13)	C14—C15—H15	118.8
O1—Co—O4	85.61 (15)	C17—C16—C15	121.9 (8)
O3—Co—O4	76.44 (13)	C17—C16—H16	119.0
C12—N11—C18	108.2 (6)	C15—C16—H16	119.0
C12—N11—H11	125.9	C16—C17—C18	115.8 (7)
C18—N11—H11	125.9	C16—C17—H17	122.1
C12—N13—C19	105.4 (5)	C18—C17—H17	122.1
C12—N13—Co	123.8 (5)	N11—C18—C17	132.3 (7)
C19—N13—Co	130.3 (3)	N11—C18—C19	104.8 (6)
C22—N21—C28	107.9 (5)	C17—C18—C19	122.8 (7)
C22—N21—H21	126.1	C14—C19—C18	120.1 (6)
C28—N21—H21	126.1	C14—C19—N13	130.9 (5)
C22—N23—C29	104.6 (5)	C18—C19—N13	109.0 (5)
C22—N23—Co	123.3 (5)	N21—C22—N23	113.8 (7)
C29—N23—Co	132.0 (4)	N21—C22—H22	123.1
C1—O1—Co	131.5 (3)	N23—C22—H22	123.1
C3—O3—Co	105.9 (3)	C25—C24—C29	118.1 (6)
C3—O3—H3A	110.0	C25—C24—H24	120.9
Co—O3—H3A	106.4	C29—C24—H24	120.9
C4—O4—Co	112.0 (3)	C24—C25—C26	119.5 (8)
C4—O5—Co^ii^	130.1 (3)	C24—C25—H25	120.3
O1—C1—O2	121.9 (6)	C26—C25—H25	120.3
O1—C1—C2	122.9 (5)	C27—C26—C25	122.8 (7)
O2—C1—C2	115.2 (6)	C27—C26—H26	118.6
C1—C2—C3	115.2 (5)	C25—C26—H26	118.6
C1—C2—H2A	108.5	C26—C27—C28	117.6 (7)
C3—C2—H2A	108.5	C26—C27—H27	121.2
C1—C2—H2B	108.5	C28—C27—H27	121.2
C3—C2—H2B	108.5	N21—C28—C27	133.4 (6)
H2A—C2—H2B	107.5	N21—C28—C29	104.8 (5)
O3—C3—C2	106.6 (4)	C27—C28—C29	121.9 (7)
O3—C3—C4	111.5 (4)	C24—C29—N23	130.9 (5)
C2—C3—C4	112.4 (4)	C24—C29—C28	120.2 (6)
O3—C3—H3	108.8	N23—C29—C28	108.9 (6)
C2—C3—H3	108.8		
			
N23—Co—N13—C12	50.3 (6)	C2—C3—C4—O5	73.8 (6)
O5^i^—Co—N13—C12	145.7 (5)	O3—C3—C4—O4	13.6 (6)
O1—Co—N13—C12	−40.6 (5)	C2—C3—C4—O4	−105.9 (5)
O4—Co—N13—C12	−126.5 (5)	C19—N13—C12—N11	0.1 (8)
N23—Co—N13—C19	−138.7 (5)	Co—N13—C12—N11	172.9 (5)
O5^i^—Co—N13—C19	−43.4 (5)	C18—N11—C12—N13	−0.1 (9)
O1—Co—N13—C19	130.3 (5)	C19—C14—C15—C16	−0.9 (10)
O4—Co—N13—C19	44.5 (5)	C14—C15—C16—C17	0.7 (12)
N13—Co—N23—C22	−121.7 (5)	C15—C16—C17—C18	−0.8 (13)
O5^i^—Co—N23—C22	145.1 (5)	C12—N11—C18—C17	−176.7 (8)
O1—Co—N23—C22	−28.7 (5)	C12—N11—C18—C19	0.1 (8)
O3—Co—N23—C22	54.5 (5)	C16—C17—C18—N11	177.6 (8)
O4—Co—N23—C22	41.4 (11)	C16—C17—C18—C19	1.2 (12)
N13—Co—N23—C29	64.3 (5)	C15—C14—C19—C18	1.3 (8)
O5^i^—Co—N23—C29	−28.9 (5)	C15—C14—C19—N13	−177.0 (6)
O1—Co—N23—C29	157.3 (5)	N11—C18—C19—C14	−178.7 (5)
O3—Co—N23—C29	−119.5 (5)	C17—C18—C19—C14	−1.5 (10)
O4—Co—N23—C29	−132.6 (8)	N11—C18—C19—N13	0.0 (7)
N23—Co—O1—C1	100.5 (5)	C17—C18—C19—N13	177.2 (7)
N13—Co—O1—C1	−163.6 (5)	C12—N13—C19—C14	178.5 (6)
O3—Co—O1—C1	7.7 (5)	Co—N13—C19—C14	6.3 (9)
O4—Co—O1—C1	−69.1 (5)	C12—N13—C19—C18	0.0 (7)
N23—Co—O3—C3	−143.1 (3)	Co—N13—C19—C18	−172.2 (4)
O5^i^—Co—O3—C3	121.8 (3)	C28—N21—C22—N23	0.6 (8)
O1—Co—O3—C3	−52.8 (3)	C29—N23—C22—N21	−0.2 (8)
O4—Co—O3—C3	34.3 (3)	Co—N23—C22—N21	−175.6 (4)
N23—Co—O4—C4	−16.0 (10)	C29—C24—C25—C26	−1.1 (11)
N13—Co—O4—C4	147.1 (3)	C24—C25—C26—C27	1.6 (13)
O5^i^—Co—O4—C4	−120.5 (3)	C25—C26—C27—C28	−0.7 (12)
O1—Co—O4—C4	54.5 (3)	C22—N21—C28—C27	177.6 (7)
O3—Co—O4—C4	−29.5 (3)	C22—N21—C28—C29	−0.8 (7)
Co—O1—C1—O2	−168.7 (4)	C26—C27—C28—N21	−178.9 (7)
Co—O1—C1—C2	12.5 (8)	C26—C27—C28—C29	−0.7 (10)
O1—C1—C2—C3	9.8 (8)	C25—C24—C29—N23	178.7 (6)
O2—C1—C2—C3	−169.0 (5)	C25—C24—C29—C28	−0.2 (10)
Co—O3—C3—C2	86.6 (4)	C22—N23—C29—C24	−179.3 (7)
Co—O3—C3—C4	−36.3 (4)	Co—N23—C29—C24	−4.5 (9)
C1—C2—C3—O3	−63.4 (6)	C22—N23—C29—C28	−0.3 (6)
C1—C2—C3—C4	58.9 (6)	Co—N23—C29—C28	174.5 (4)
Co^ii^—O5—C4—O4	−9.6 (7)	N21—C28—C29—C24	179.8 (6)
Co^ii^—O5—C4—C3	170.8 (3)	C27—C28—C29—C24	1.2 (9)
Co—O4—C4—O5	−162.3 (4)	N21—C28—C29—N23	0.7 (6)
Co—O4—C4—C3	17.4 (5)	C27—C28—C29—N23	−178.0 (6)
O3—C3—C4—O5	−166.7 (4)		

Symmetry codes: (i) *y*−1/2, −*x*+1, −*z*+1; (ii) −*y*+1, *x*+1/2, −*z*+1.

### Hydrogen-bond geometry (Å, °)

**Table:**

*D*—H···*A*	*D*—H	H···*A*	*D*···*A*	*D*—H···*A*
N11—H11···O3WA	0.86	2.18	3.00 (4)	159
N11—H11···O5WA^iii^	0.86	2.10	2.93 (4)	163
N21—H21···O1^iv^	0.86	2.57	3.258 (7)	138
N21—H21···O2^iv^	0.86	2.07	2.901 (8)	163
O3—H3A···O4^i^	0.92	1.78	2.645 (5)	155

Symmetry codes: (iii) *y*, −*x*+3/2, *z*; (iv) −*y*+3/2, *x*, *z*; (i) *y*−1/2, −*x*+1, −*z*+1.
